# [^68^Ga]Pentixafor PET/MR imaging of chemokine receptor 4 expression in the human carotid artery

**DOI:** 10.1007/s00259-019-04322-7

**Published:** 2019-04-19

**Authors:** Xiang Li, Wei Yu, Tim Wollenweber, Xia Lu, Yongxiang Wei, Dietrich Beitzke, Wolfgang Wadsak, Saskia Kropf, Hans J. Wester, Alexander R. Haug, Xiaoli Zhang, Marcus Hacker

**Affiliations:** 1 Division of Nuclear Medicine, Department of Biomedical Imaging and Image-Guided Therapy, Medical University of Vienna, Währinger Gürtel 18-20, 1090 Vienna, Austria; 2 Department of Radiology, Beijing Anzhen Hospital, Capital Medical University, Beijing, China; 3 Department of Nuclear Medicine, Beijing Anzhen Hospital, Capital Medical University, Anzhen Street No. 2, Beijing, 100029 China; 4 Division of Cardiovascular and Interventional Radiology, Department of Biomedical Imaging and Image-guided Therapy, Medical University of Vienna, Vienna, Austria; 5 Center for Biomarker Research in Medicine, CBmed, Graz, Austria; 6 Scintomics GmbH, Fürstenfeldbruck, Germany; 7 Department of Radiopharmaceutical Chemistry, Technische Universität München, Garching, Germany

**Keywords:** Atherosclerosis, [^68^Ga]Pentixafor, CXCR4, PET/MRI, Carotid artery

## Abstract

**Purpose:**

Type 4 chemokine receptor (CXCR4) plays an important role in immune cell migration during the atherosclerosis progression. We aimed to evaluate [^68^Ga]Pentixafor positron emission tomography (PET) in combination magnetic resonance imaging (MRI) for in vivo quantification of CXCR4 expression in carotid plaques.

**Methods:**

Seventy-two patients with lymphoma were prospectively scheduled for whole body [^68^Ga]Pentixafor PET/MRI with an additional T2-weighted carotid sequence. Volumes of interest (VOIs) were drawn along the carotid bifurcation regions, and the maximum tissue-to-blood ratios (TBR) of [^68^Ga]Pentixafor uptake were calculated. Lesions were categorized into non-eccentric (*n* = 27), mild eccentric (*n* = 67), moderately (*n* = 41) and severely (*n* = 19) eccentric carotid atherosclerosis. A different cohort of symptomatic patients (*n* = 10) with carotid stenosis scheduled for thrombendarterectomy (TEA) was separately imaged with 3T MRI with dedicated plaque sequences (time of flight, T1-, and T2-weighted). MRI findings were correlated with histochemical assessment of intact carotid plaques.

**Results:**

At hybrid PET/MRI, we observed significantly increased [^68^Ga]Pentixafor uptake in mildly (mean TBR_max_ = 1.57 ± 0.27, mean SUV_max_ = 2.51 ± 0.39), moderately (mean TBR_max_ = 1.64 ± 0.37, mean SUV_max_ = 2.61 ± 0.55) and severely eccentric carotids (mean TBR_max_ = 1.55 ± 0.26, mean SUV_max_ = 2.40 ± 0.44) as compared to non-eccentric carotids (mean TBR_max_ = 1.29 ± 0.21, mean SUV_max_ = 1.77 ± 0.42) (*p* ≤ 0.05). Histological findings from TEA confirmed that prominent CXCR4 expression was localized within inflamed atheromas and preatheromas. Co-localization of cellular CXCR4 and CD68 expression in the plaque was observed by immunofluorescence staining.

**Conclusions:**

In vivo evaluation of CXCR4 expression in carotid atherosclerotic lesions is feasible using [^68^Ga]Pentixafor PET/MRI. In atherosclerotic plaque tissue, CXCR4 expression might be used as a surrogate marker for inflammatory atherosclerosis.

## Introduction

Atherosclerotic plaques develop from a thickening of the vascular wall due to progressive accumulation of inflammatory cells and oxidized lipid components in the arterial intima [1]. The rupture of an atherosclerotic plaque is the essential cause of sudden heart attack and stroke [2]. The challenge in clinical practice is to identify and treat vulnerable plaque lesions before severe cardiovascular events occur [3]. High-resolution MRI scans, including time of flight (TOF) and T1- and T2-weighted imaging due to the high contrast to soft tissue, was shown to be sensitive for the characterization of atherosclerosis [4], including identification of lipid components, such as intra-plaque hemorrhage [5], fibrous cap [6], calcification or the lipid core [7]. Plaque lesions were also classified into different types by MRI in relation to histological examinations [4]. In addition to MRI anatomical imaging of plaques, the use of specific radiotracers to measure intra-plaque inflammatory targets or atherosclerosis progression [8] by PET has become a major complementary approach for MRI diagnostics [9].

2-[^18^F]fluoro-2-deoxy-*D*-glucose ([^18^F]FDG) is the most widely investigated PET tracer for plaque inflammation imaging. However, the relatively low specificity of ^18^F-FDG to macrophages is a well-known limitation for plaque imaging [8]. Thus, to discover more alternative inflammation-specific radiotracers has become the primary objective in PET diagnostics of atherosclerosis. The CXC ligand (CXCL) and the 12/CXC receptor (CXCR4) were demonstrated to participate in the recruitment of leukocytes into the vessel wall [10, 11]. CXCR4 cellular expression gradually accumulates during plaque progression in human atherosclerotic lesions [12]. A pro-inflammatory function of CXCR4 was demonstrated for the recruitment of progressively infiltrating monocytes into the vascular intima by binding of the macrophage migration inhibitory factor (MIF) [13, 14]. The expression of CXCR4 is also significantly upregulated under hypoxic conditions within atherosclerosis-associated cells [15].

Recently, [^68^Ga]Pentixafor has been introduced as a novel PET tracer that specifically binds to the CXCR4 [16]. Moreover, [^68^Ga]Pentixafor PET has been shown to be sensitive and reproducible for the characterization of atherosclerotic lesions [17–19]. Nevertheless, systematic quantification of the vascular uptake of [^68^Ga]Pentixafor in atherosclerotic lesions still requires an accurate delineation of plaques. Integrated PET-MRI hybrid imaging is an emerging new tool that has shown great potential in plaque imaging [20].

The purpose of the present study was to quantify and classify CXCR4 expression by the use of [^68^Ga]Pentixafor PET/MRI within human carotid lesions. Furthermore, MRI features were correlated to immunohistochemical findings.

## Methods and materials

### Patient population

One hundred consecutive patients scheduled for biopsy of treatment naive mucosa-associated lymphoid tissue (MALT) lymphoma lesions were prospectively included in a clinical trial assessing the prognostic impact of ^68^Ga-Pentixafor uptake between August 2016 and June 2018. All patients underwent [^68^Ga]-Pentixafor PET/MRI with additional T2-weighted MR imaging with a black-blood sequence of the neck and thorax region to determine the luminal eccentric level of carotid arteries. Baseline clinical data are summarized in Table 1. The clinical institutional review board of the Medical University Vienna approved this study and all patients provided written, informed consent.

**Table 1:** Baseline characteristics of the [^68^Ga]Pentixafor PET/MRI study cohort

Patients (N = 72)	
Demographics	
Age, mean ± SD	61.8 ± 12.7
Men, *n* (%)	45 (62.5)
Body-mass index (kg/m^2^), mean ± SD	26.8 ± 4.0
Risk factors, *n* (%)	
Hypertension	24 (33.3)
Diabetes type II	8 (11.1)
Hypercholesterolemia	8 (11.1)
Smoking	22 (30.6)
Medication for cardiovascular diseases, *n* (%)	
Angiotensin-converting-enzyme (ACE) inhibitor	5 (6.9)
Beta blocker	11 (15.3)
Calcium antagonists	6 (8.3)
Diuretic therapy	12 (16.6)
Aspirin	2 (2.8)

**Fig. 1:**
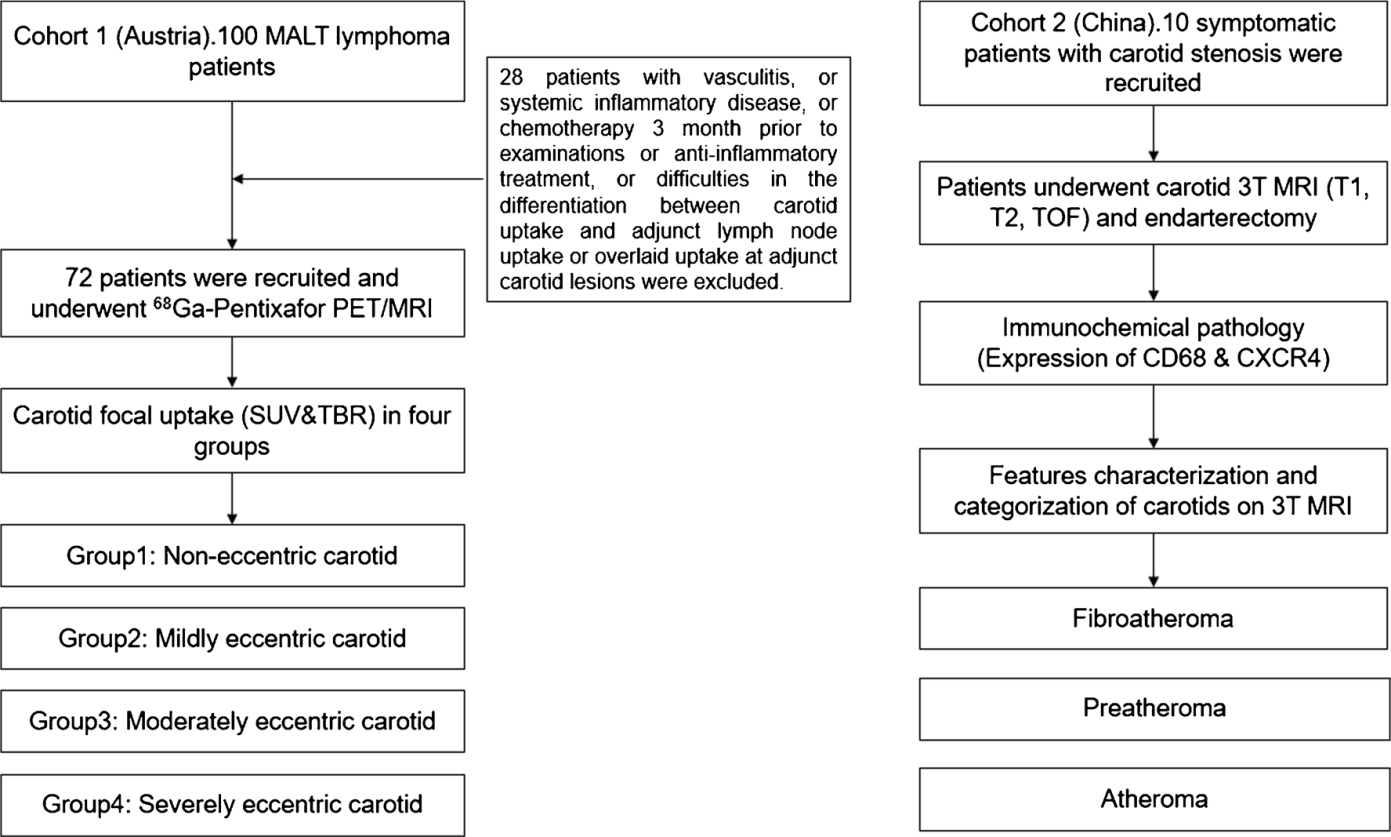
Study flowchart. MALT, mucosa-associated lymphoid tissue; SUV, standardized uptake value; TBR, target to background ratio; TOF, time of flight

In addition, ten symptomatic patients with carotid stenosis scheduled for carotid endarterectomy were recruited between December 2017 and March 2018 in Beijing Anzhen Hospital for MRI examinations and histological analysis of tissue specimens (Fig. 1). The institutional review board of Beijing Anzhen Hospital (China) approved this study. Patient age, gender, body mass index (BMI), and common cardiovascular risk factors, including smoking, hypertension, and hypercholesterolemia were documented.

### PET/MRI in lymphoma patients

Lymphoma patients underwent [^68^Ga]Pentixafor whole body PET/MR imaging (Biograph mMR; Siemens, Erlangen, Germany) (171 ± 33 MBq [range: 78–251 MBq]) to assess CXCR4 expression for the purpose of lymphoma staging and restaging. To determine the luminal eccentric level of carotid arteries, an additional T2-weighted MR black-blood sequence over the neck and thorax region was performed with an integrated radiofrequency coil and a multi-station protocol with a slice thickness of 2 mm. Attenuation correction was performed using the implemented standard four-compartment model attenuation map, calculated from a Dixon-based VIBE (volumetric interpolated breath-hold examination) sequence. A 3D ordinary Poisson-ordered subset expectation maximization (OP-OSEM) algorithm, with PSF correction and three iterations and 21 subsets, was used for reconstruction. The image matrix size was 172 × 172 (pixel size 4.2 mm). The images were smoothed with a 3-mm full-width-at-half-maximum (FWHM) Gaussian filter.

The reconstructed PET/MRI data were analyzed, and carotid lesions were categorized based on the luminal obstruction level on the cross-sectional appearance of carotid artery. Subsequently, the presence of [^68^Ga]Pentixafor uptake was determined as previously reported [17, 20, 21]. Briefly, all axial PET image slices were inspected visually along the internal carotid arterial segments. Maximum standardized uptake values (SUV_max_) were derived from 3D VOIs, which were drawn at the visualized atherosclerotic wall uptake or the non-eccentric carotid bifurcation. As a reference, SUV_bloodpool_ was calculated as the SUV_mean_ of three VOIs within the lumen of the vena cava. The TBR_max_ of carotid lesions was calculated by the respective SUV_max_ values corrected for background blood-pool activity [17, 22] .

### 3T-MRI in symptomatic patients with carotid stenoses for correlation of atherosclerotic plaque anatomy with histology

To characterize the MR-morphology of carotid plaques in relation to histology (N = 10), 3T MRI (MAGNETOM Verio 3T, SIEMENS) with a dual surface coil positioned on top of the mandibular angles was applied in a different cohort of symptomatic patients with carotid stenosis. Two contrast-weighted MR images in the transverse plane were acquired, including T1-weighted and T2-weighted images. T1-weighted MRI was acquired with a turbo spin echo (TSE) sequence or a black-blood sequence, with a two-dimensional fast-spin echo (FSE). Imaging parameters included TR/TE = 800/9.3 ms; T2-weighted: FSE, cardiac-gated, 40 ms for echo TE, thickness of 2 mm, 256 × 256 matrix, NEX2. Fat suppression was used for T1- and T2-weighted images. In-plane resolution was 1 mm. For 3D-TOF scan: TR/TE 23/3.8 ms, FOV 13 cm, thickness of 1 mm, 256 × 256 matrix, NEX2. Two experienced radiologists characterized the carotid intraplaque components. T1-weighted, T2-weighted, and TOF MR transversal images from 3T MRI of TEA patients were correlated and categorized according to carotid histological specimen sections. Morphological features, including calcification, fibrous tissue, lipid deposition, and hemorrhage, were assessed and correlated with histological and immunohistochemical findings.

### Visual classification of carotid atherosclerotic plaques

**Fig. 2:**
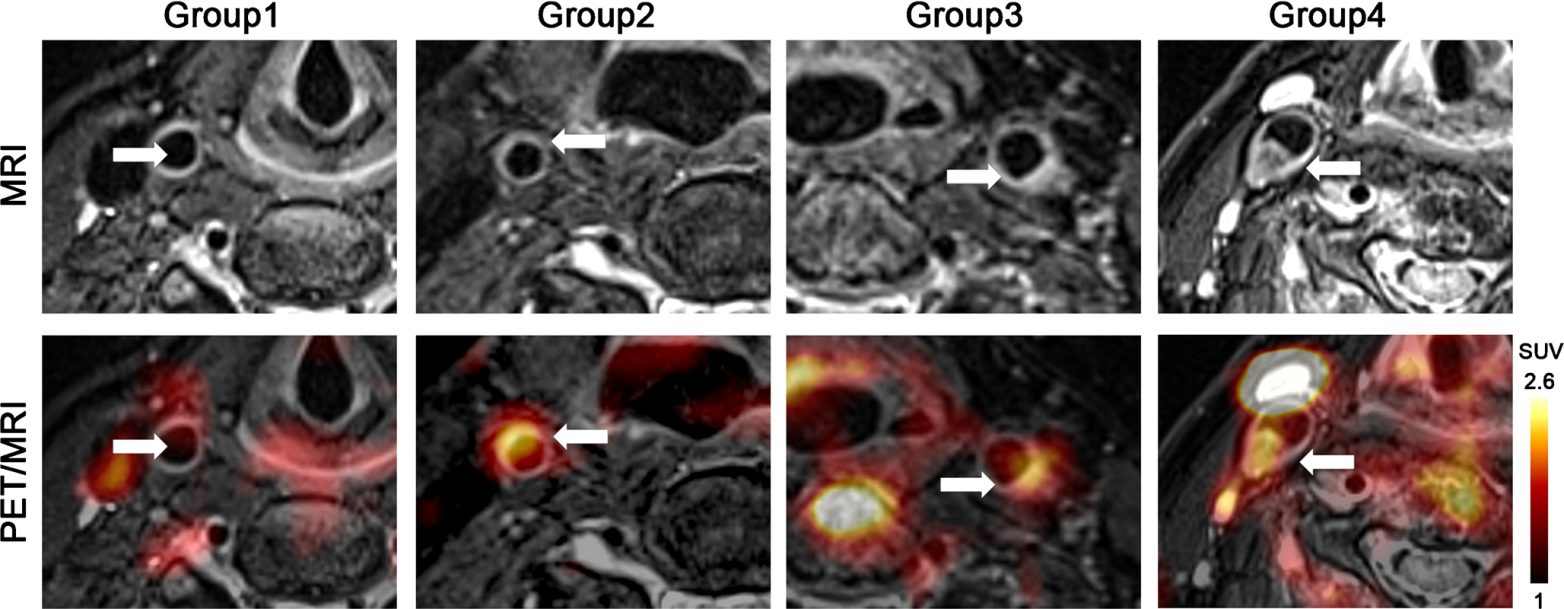
Example transaxial [^68^Ga]Pentixafor PET/MRI images of carotid lesions within different groups. Focal uptake was observed in a mildly atherosclerotic carotid artery showing a slightly eccentric thickening (Group 1) and in a moderately (Group 3) and severely (Group 4) atherosclerotic carotid showing significant eccentric thickening, increased tracer uptake was absent at non-significantly eccentric control carotid (Group 1). *Arrows* indicate the arterial regions of interest

Atherosclerotic carotid lesions (N = 154, 2.14 lesions/patient) were visually assessed on a hybrid PET/MRI system and semi-quantitatively classified into four groups with luminal obstructive features on T2-weighted carotid MRI: Group 1, Non-eccentric carotids (non-significant luminal obstruction); Group 2, mild carotid atherosclerosis with slight eccentric thickening of the arterial wall (cross-sectional luminal obstruction ≤10%); Group 3, moderately eccentric carotid atherosclerosis with intermediate thickening of the arterial wall (cross-sectional luminal obstruction >10%); Group 4, severely eccentric carotid atherosclerosis with significant thickening of the arterial wall (cross-sectional luminal obstruction ≥25%). Representative transaxial ^68^Ga-Pentixafor PET/MRI images of patients with different thickening level lesions are shown in Fig. 2.

### Immunohistochemistry

Plaque build-up was obtained from carotid arteries in patients (N = 10) who underwent carotid endarterectomy and 3T MRI plaque imaging. For general morphology, a standard elastin Masson’s trichrome stain was performed on carotid plaque segments to visualize collagen tissue within atherosclerotic lesions. Immunohistochemistry was also performed to detect CXCR4 expression in the carotid sections of the representative plaque lesions. Briefly, plaque tissue was dried overnight and fixed in cold acetone for 10 min and washed for staining. Tissues were then incubated with primary anti-CXCR4 (ab124824, abcam) and anti-CD68 (ab955, abcam) antibodies at 4 °C overnight, labelled with polymer-HRP anti-rabbit antibody, and visualized with 3.3′-diaminobenzindine (DAB) (Sigma-Aldrich). Subsequently, tissue slides were dipped in de-stain solution and then washed in tap water for 5 min. Finally, slides were dehydrated in alcohol with a graduated concentration (30% to 70% to 100%). Additionally, immunofluorescence staining was performed on 5-μm-thick plaque sections and double-stained with primary anti-CXCR4 (1:50, ab1670, abcam) and anti-CD3 antibody (1:100, ab5690, abcam), as well primary anti-CD68 antibody (1:50, ab955, abcam) respectively. 4′,6-diamidino-2-phenylindole (DAPI) was used for nuclear imaging. All slides were mounted for microscopic imaging after incubation with secondary antibody. Cellular counting was performed under Zeiss Axioplan light microscope at ×200 magnification.

### Statistical analysis

To compare [^68^Ga]Pentixafor uptake between categorized lesions, the mean ± SD of TBR_max_ and SUV_max_ values of each group were calculated and subsequently compared using one-way ANOVA. A Games-Howell post hoc test was performed to confirm when differences occurred. Mean TBR_max_ and SUV_max_ values were additionally calculated from all PET assessed carotid lesions per patient to correlate (Pearson correlation coefficients) with age, gender, body mass index (BMI), and common cardiovascular risk factors, including smoking, hypertension, and hypercholesterolemia. All statistical analyses were performed with the SPSS version 19 (SPSS Inc., Chicago, IL). *P*-values <0.05 were considered statistically significant. Percentages of CXCR4 positive areas to total plaque areas were calculated with a commercial software package, which quantifies the intensity of positive signals in each field (Image J, version 1.8.0_112).

## Results

### Patients

Patients with vasculitis, systemic inflammatory disease, record of chemotherapy 3 months prior to examinations or anti-inflammatory treatment were excluded. Patients were also excluded for the final evaluation in case of difficulties in the differentiation between carotid uptake and adjacent lymph node uptake or overlaid uptake with adjacent carotid lesions (Fig. 1). Patient characteristics of the remaining 72 patients are presented in Table 1.

### [^68^Ga]Pentixafor carotid PET/MRI

**Fig. 3:**
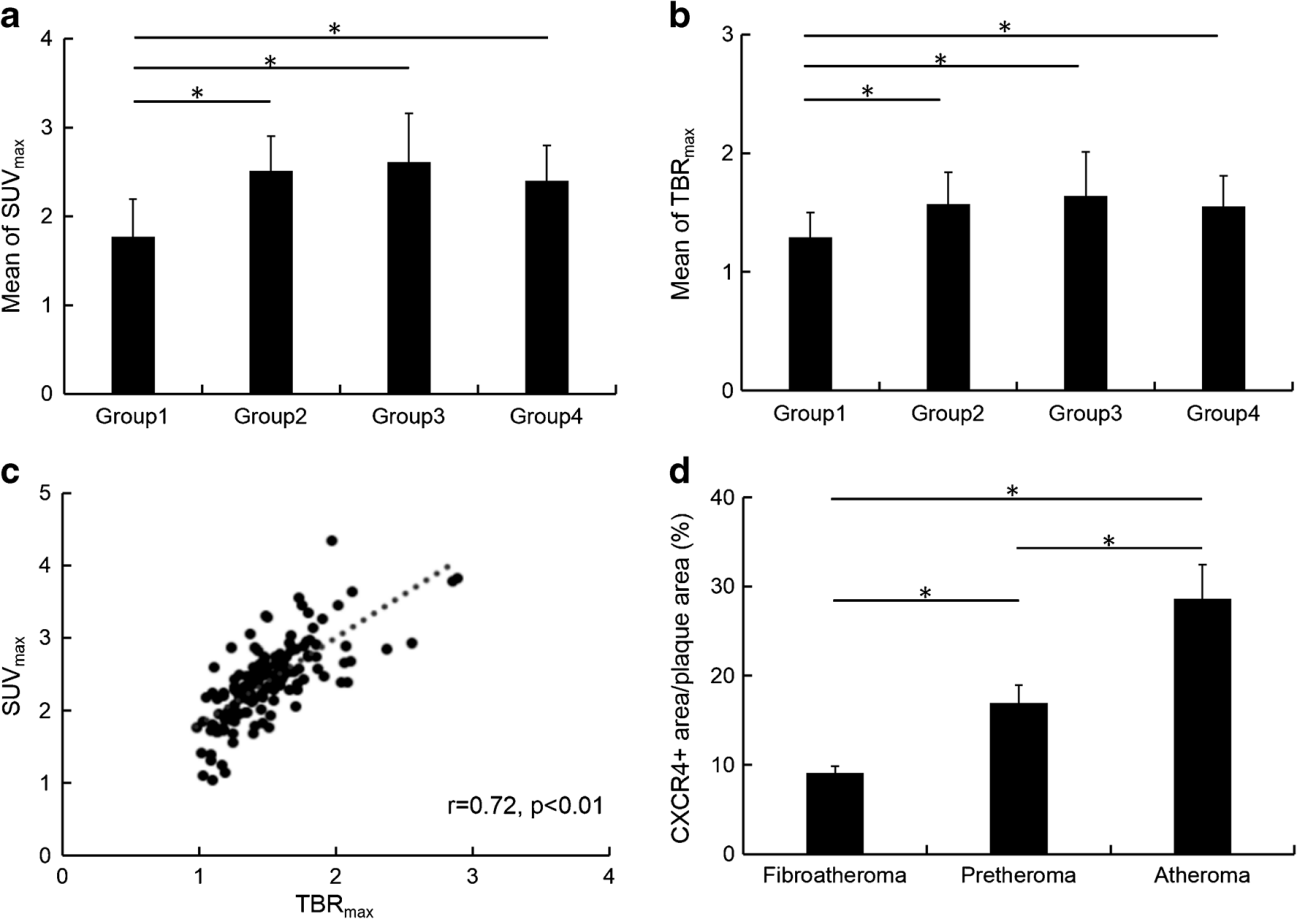
**a** and **b** [^68^Ga]Pentixafor uptake ratios (mean of TBR_max_ and SUV_max_) of categorized atherosclerotic lesions. In group 1, non-obstructive carotids (N = 27), uptake was significantly lower (*p < 0.05) compared to group 2 (mildly eccentric carotid atherosclerosis, N = 67), group 3 (moderately eccentric carotid atherosclerosis, N = 41) and group 4 (severely eccentric carotid atherosclerosis, N = 19). There was no significant difference between other groups. **c** Linear relationship between TBR_max_ and SUV_max_ in all lesions (Pearson’s r = 0.72, *p < 0.01). **d** The percentages (r) of CXCR4+ plaque area/ total plaque area in cross-sections were assessed in categorized carotid lesions as 9.08 ± 0.74% for fibroatheroma (N = 2), 16.95 ± 2.01% for preatheroma (N = 3) and 28.58 ± 3.83% for inflamed atheroma (N = 5). * Significance level of *p* ≤ 0.05

[^68^Ga]Pentixafor uptake in patient group 1 (non-eccentric carotids, mean TBR_max_ = 1.29 ± 0.21; mean SUV_max_ = 1.77 ± 0.42, N = 27) was significantly lower than in group 2 (mildly eccentric carotid atherosclerotic lesions, mean TBR_max_ = 1.57 ± 0.27 and mean SUV_max_ = 2.51 ± 0.39; N = 67), group 3 (moderately eccentric atherosclerotic carotid lesions, mean TBR_max_ = 1.64 ± 0.37 and mean SUV_max_ = 2.61 ± 0.55; N = 41) and group 4 (severely eccentric atherosclerotic carotid lesions, mean TBR_max_ = 1.55 ± 0.26 and mean SUV_max_ = 2.40 ± 0.44; N = 19) (*p* < 0.05) (Fig. 3a, b). [^68^Ga]Pentixafor uptake did not statistically differ between groups 2, 3 and 4. There was a significant correlation found between [^68^Ga]Pentixafor uptake (TBR_max_ and SUV_max_) and the prevalence of hypertension (Pearson’s r = 0.27/ Pearson’s r = 0.35; *p* < 0.05) and diabetes type II (Pearson’s r = 0.27/ Pearson’s r = 0.35; *p* < 0.05). SUV_max_ and TBR_max_ were also significantly correlated (Pearson’s r = 0.72, *p* < 0.01) (Fig. 3c).

### Association between carotid MRI and histology

**Fig. 4:**
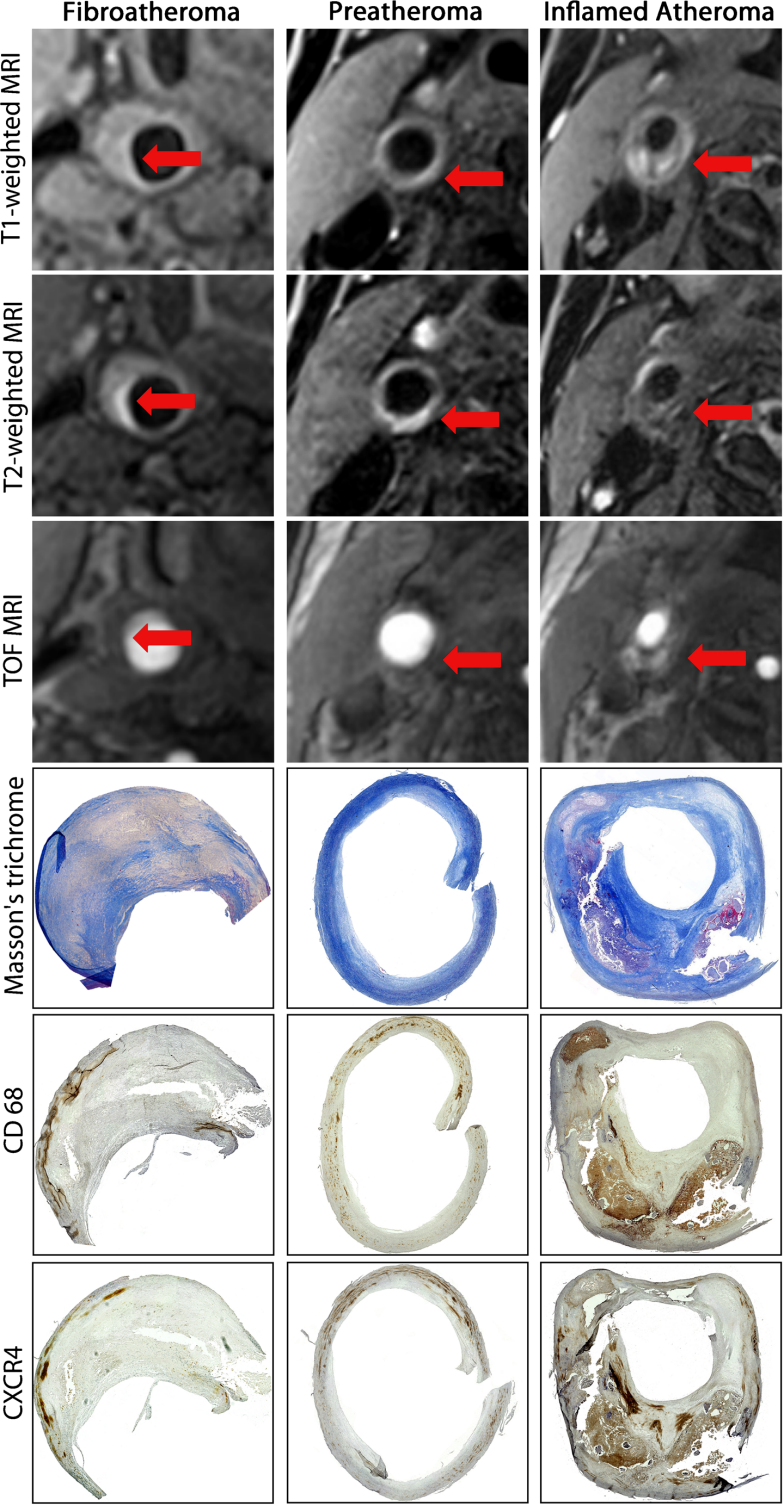
Multi-sequenced MRI of the internal carotid arteries, including time-of-flight (TOF), T1-, and T2-weighted protocols. Corresponding histology (collagen Masson’s Trichrome stain) and immunohistochemistry (CD68 and CXCR4) examination of excised carotid plaques were compared with MRI. *Arrows* indicate the carotid plaque regions of interests in cross-section views, which were correlated with immunohistochemistry results. Relative lower CD68 expression and CXCR4 expression was observed within fibrous tissue (*first column*) and increased CXCR4 expression along with increased infiltrated monocytes/macrophages, as indicated by CD68, was detected in preatheroma (*second column*) during early plaque formation, particularly prominent in atheroma with thin fibrotic cap (*third column*)

**Fig. 5:**
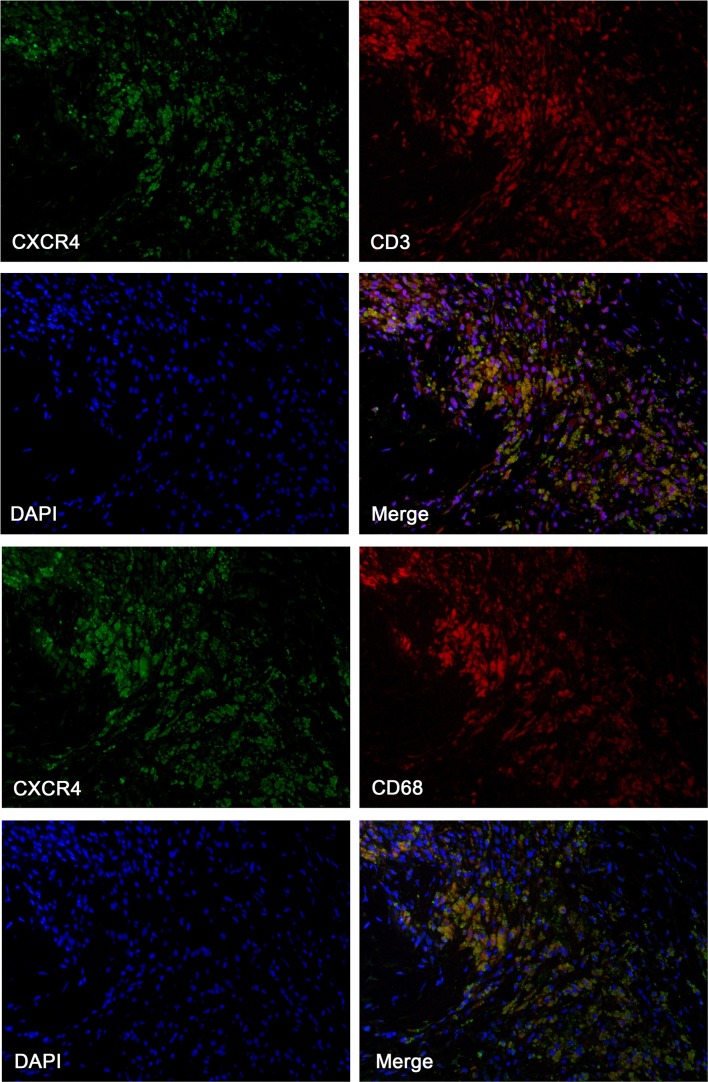
Representative immunofluorescence staining of an excised carotid plaque. CXCR4 expression (*green*) in excised carotid plaque, CD3 staining (*left red*) for expression of T cells, CD68 staining (*left red*) for expression of macrophages and nuclear counterstaining with DAPI (*blue*), 4′,6-diamidino-2-phenylindole. CXCR4 expression was partly co-localized with CD3-positive cells and mostly co-localized with CD68-positive cells

Carotid MRI anatomical findings were correlated with immunohistochemistry results. We observed that there was prominent CXCR4 expression within carotid atheromas with a large necrotic core, whereas the regional CXCR4 expression occurred mainly in the necrotic cores and surrounding tissues, in which it was co-localized with infiltrated macrophages, as determined by positive CD68 expression. Nevertheless, CXCR4 expression was relatively lower in non-inflamed firboatheromas (CXCR4+ area/plaque area = 9.08 ± 0.74%, N = 2), and increased in pretheromas (CXCR4+ area/ plaque area = 16.95 ± 2.01%, N = 3) (*p* < 0.05), and most elevated in inflamed atheroma (CXCR4+ area/plaque area = 28.58 ± 3.83%, N = 5) (*p* < 0.05) (Fig. 3d). We also observed upregulated expression of CXCR4 at inflamed preatheromas, which occurred at a relatively early phase of atherosclerosis with infiltrated macrophages. In this present study, we discovered that the elevated CXCR4 expression in the atheromas was particularly localized in adipose tissue (Fig. 4), and CXCR4 expression mostly co-localized with CD3 (T-cells) and CD68 expression (macrophages/monocytes) by immunofluorescence staining (Fig. 5).

## Discussion

In the present study we related CXCR4 expression as quantified by [^68^Ga]Pentixafor PET to atherosclerotic lesions with different grades of stenoses using T2-weighted MRI. Remarkably, we observed relatively low [^68^Ga]Pentixafor carotid uptake in non-stenosis lesions compared to eccentric atherosclerotic lesions, which showed significantly higher CXCR4 expression. In a previously published PET/MRI study, higher focal [^68^Ga]Pentixafor uptake was already described in significant (>50%) as compared to non-significant carotid stenosis (<30%) [18]. A further retrospective study assessed [^68^Ga]Pentixafor large arterial PET uptake in non-cardiovascular patients. That study reported that 33.5% of hot lesions on [^68^Ga]Pentixafor showed calcification, while only 7.3% calcified lesions showed increased [^68^Ga]Pentixafor uptake [22].

In addition to these results, we correlated histology examinations of typical plaque lesions with corresponding non-invasive carotid characterization by MRI, which revealed intensive CXCR4 expression in inflamed atheroma and preatheroma, but weak expression in macrophage-poor fibrotic lesions. This is again in line with previous studies. Hyafil et al. reported from a preclinical study that the CXCR4 protein was expressed in macrophage-rich arterial tissues, and demonstrated great potential for [^68^Ga]Pentixafor PET to specifically recognize inflamed atherosclerotic lesions [18]. In another recent study, Derlin et al. demonstrated that the [^68^Ga]Pentixafor PET signal intensively accumulated in culprit coronary atherosclerotic plaques of patients with recent acute myocardial infarction [19].

The underlying function and expression of the CXCL12-CXCR4 axis during native atherosclerosis progression is complex and still remains unclear. Previous studies identified the CXCL12-CXCR4 axis to be responsible for progenitor cell recruitment into injured peripheral tissue, which could serve as biomarkers to predict pro-inflammatory leukocyte mobilization within cardiovascular diseases [10]. In view of the CXCR4 protein in atherosclerosis, the function and expression of two alternative ligands of CXCR4, MIF and CXCL12 were summarized and compared [14]. In a recent study [23], Merkelbach and colleagues demonstrated significantly higher expression of CXCR4 within culprit human plaques in comparison to control lesions. Remarkably, they also presented that the cellular expression of CXCR4 was specifically higher in CD68-positive macrophages in comparison to CD45-positive leukocytes and SM-actin-positive smooth muscle cells. Thus, CXCR4-specific expression might be a response to an inflammatory stimulus, in close association with pro-atherogenic activity. In this present study, we observed co-localization of atherosclerotic CXCR4 expression with T cells (CD3) and macrophages (CD68). This might indicate increasing recruitment of leukocytes by interaction with CXCR4, which is responsible for pro-inflammatory reactions during atherosclerosis progression. Thus, CXCR4 could be identified as a pro-inflammatory mediator within the immune system; in particular, its expression in atherosclerotic lesions mediates the arrest of leukocytes in the intimal layer of vessels, and the process is up-regulated in response to activated inflammation [10, 13, 24, 25]. A pioneering study showed that lesion up-regulation of MIF within plaques is closely related to an increased vulnerability through the weakening of the fibrous cap [26]; and inhibition of MIF could induce attenuation of foam cell formation, macrophage infiltration [27], and regression of established plaque lesions [28]. These findings suggest that the CXCR4 protein might implement atherosclerotic plaque progression and mediate its destabilization. In contrast, the function of CXCL12 is mainly related to homing progenitors in the bone marrow and migration of the immune cells into the periphery. Indeed, the prevention of the release of progenitor cells and leukocytes from bone marrow into the blood circulation seemed to demonstrate a protective role for attenuated atherosclerosis progression [29].

Thus, [^68^Ga]Pentixafor demonstrated its potential for atherosclerotic plaque imaging not only because the CXCR4 protein is expressed at various cell types related to atherogenesis [12, 30–34] and particularly the intensive co-localization of CXCR4 expression and macrophages ensures an adequate radio-signal for PET imaging, but also importantly, compared to ^18^F-FDG, low myocardial uptake of ^68^Ga-pentixfor could enable coronary imaging without specific patient preparation [19].

Overall, on-site CXCR4 proteins were specifically expressed within inflamed lesions. The overexpression of the vascular CXCR4 protein might, thus, indicate either enhanced infiltration of immune cells or healing of injured/inflamed vasculature, so that in vivo quantification of CXCR4 might be used as a transient inflammation biomarker for the molecular imaging of atherosclerosis, which could provide real-time information about on-site inflammatory activity.

Nevertheless, systematic analyses of the exact functional expression of CXCR4 during atherosclerosis progression are required in order to offer robust cellular validation of CXCR4 as a specific marker of atherosclerosis.

### Limitations

There are several limitations of this study. We prospectively included oncological patients in this clinical PET/MRI study, so that the major findings might not be well-applicable to a non-oncological population. PET/MRI did not include high resolution plaque sequences, which has limited the anatomical characterization of the arterial wall. Furthermore, no histological reference was obtained in accordance with PET/MRI imaging findings, and the tracer uptake by activated immune cells in vulnerable plaques with acute/subacute haemorrhage was not obtained. Thus, further investigation should involve larger, longitudinal examinations of symptomatic patients, with clinical outcomes, particularly to monitor [^68^Ga]Pentixafor accumulation during the progression of early, subclinical atherosclerosis. The high positron energy of ^68^Ga could have affected the precise anatomical localization, and partial volume effects could have affected the PET quantification in small carotid plaque lesions. Additionally, it still remains unclear if the CXCR4 expression from histologically assessed carotid plaques could be translated into clinical [^68^Ga]Pentixafor PET imaging.

## Conclusion

[^68^Ga]Pentixafor uptake was significantly increased in eccentric carotid artery, and most elevated at eccentric carotid atherosclerosis. Thus, [^68^Ga]Pentixafor PET/MRI has a potential to noninvasively evaluate atherosclerotic lesions by combining morphological plaque evaluation with quantifying CXCR4 expression which is related to inflammatory activity.
